# Supplementary material for: Towards Intraoperative Quantification of Atrial Fibrosis Using Light-Scattering Spectroscopy and Convolutional Neural Networks
Source: Sensors (Basel). 2021 Sep 9;21(18):6033. doi: 10.3390/s21186033 (PMC8471003; doi:10.3390/s21186033)
Supplement: Supplementary file 1 [file sensors-21-06033-s001.zip › sensors-1366626-supplementary.pdf]

Towards Intraoperative Quantification of Atrial Fibrosis Using Light Scattering Spectroscopy and Convolutional Neural Networks

Nathan J. Knighton, Brian K. Cottle, Bailey E. B. Kelson, Robert W. Hitchcock, and Frank B. Sachse

Supplementary Information

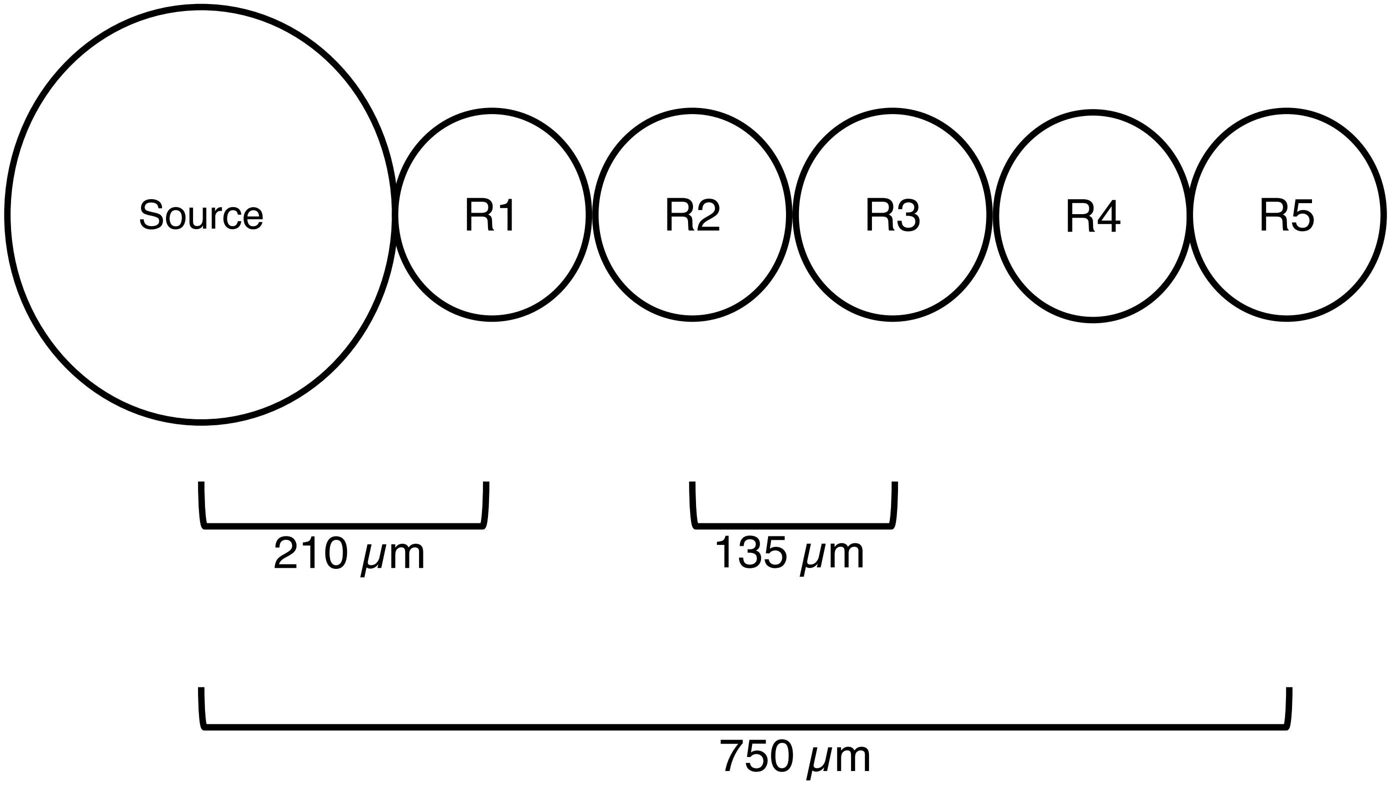

Figure S1. Arrangement of collection and illumination fibers. Spectra were collected from the fibers R1 to R5 with (center-center) distances of 210, 345, 480, 615, and 750 μm, respectively, from the illumination fiber.

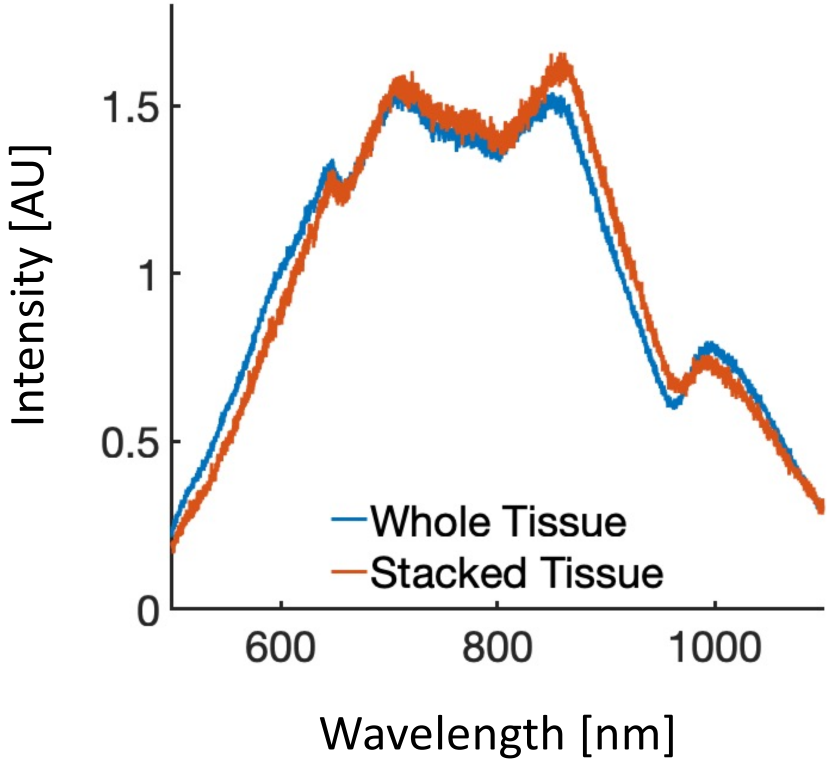

Figure S2. Example spectra acquired from epicardial surface of canine ventricle (unsectioned) and constructs of sections of ventricular tissue. Spectra were normalized.

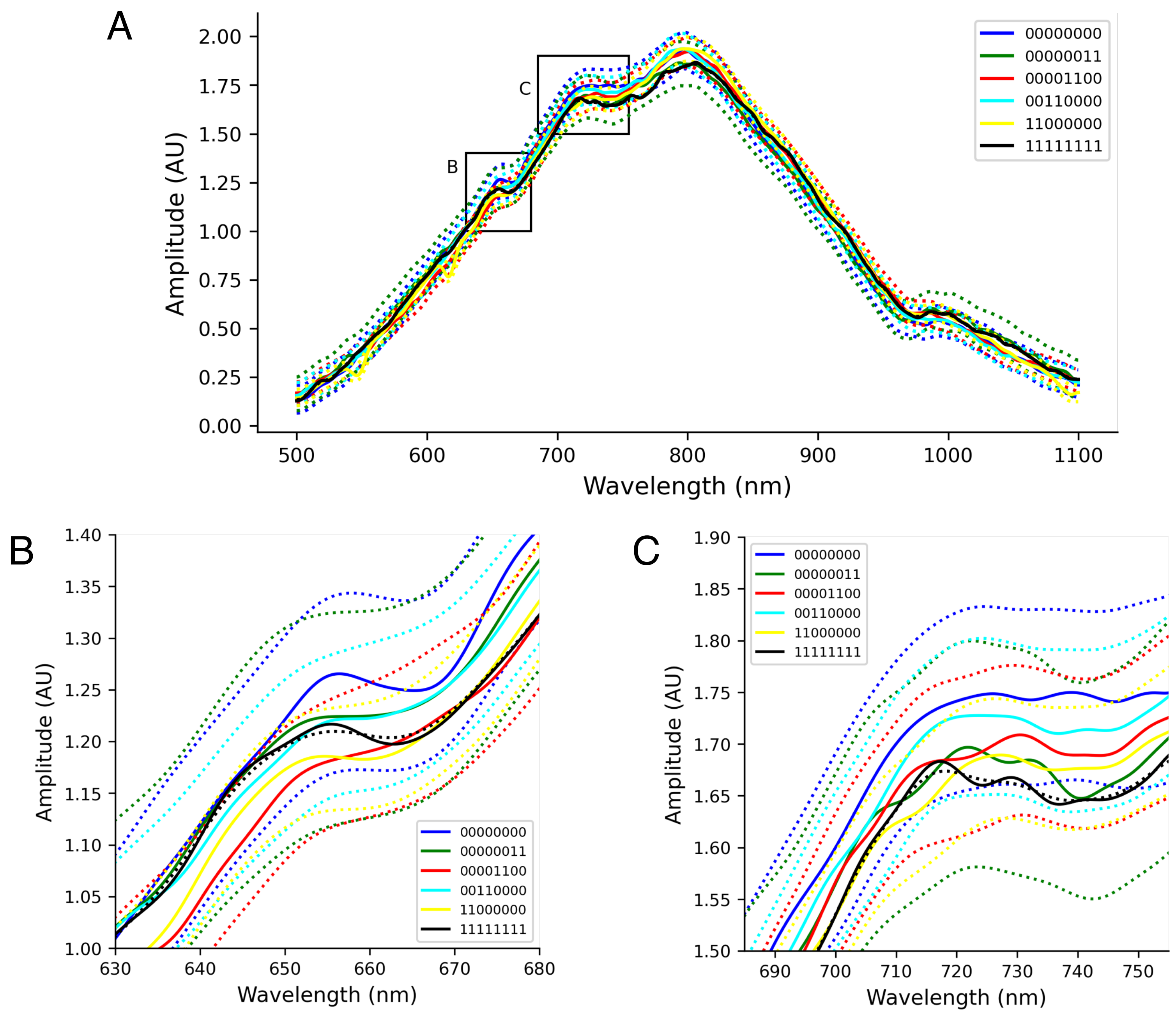

Figure S3. Spectra for training for assessment of depth detection. (A) Mean (bold line) and standard deviation (dotted line) of spectra. Spectra were normalized. The spectra also were filtered to improve visualization using a 1D gaussian kernel with standard deviation of 20 measures (~3.3 nm). (B, C) Zoom into marked regions in (A).

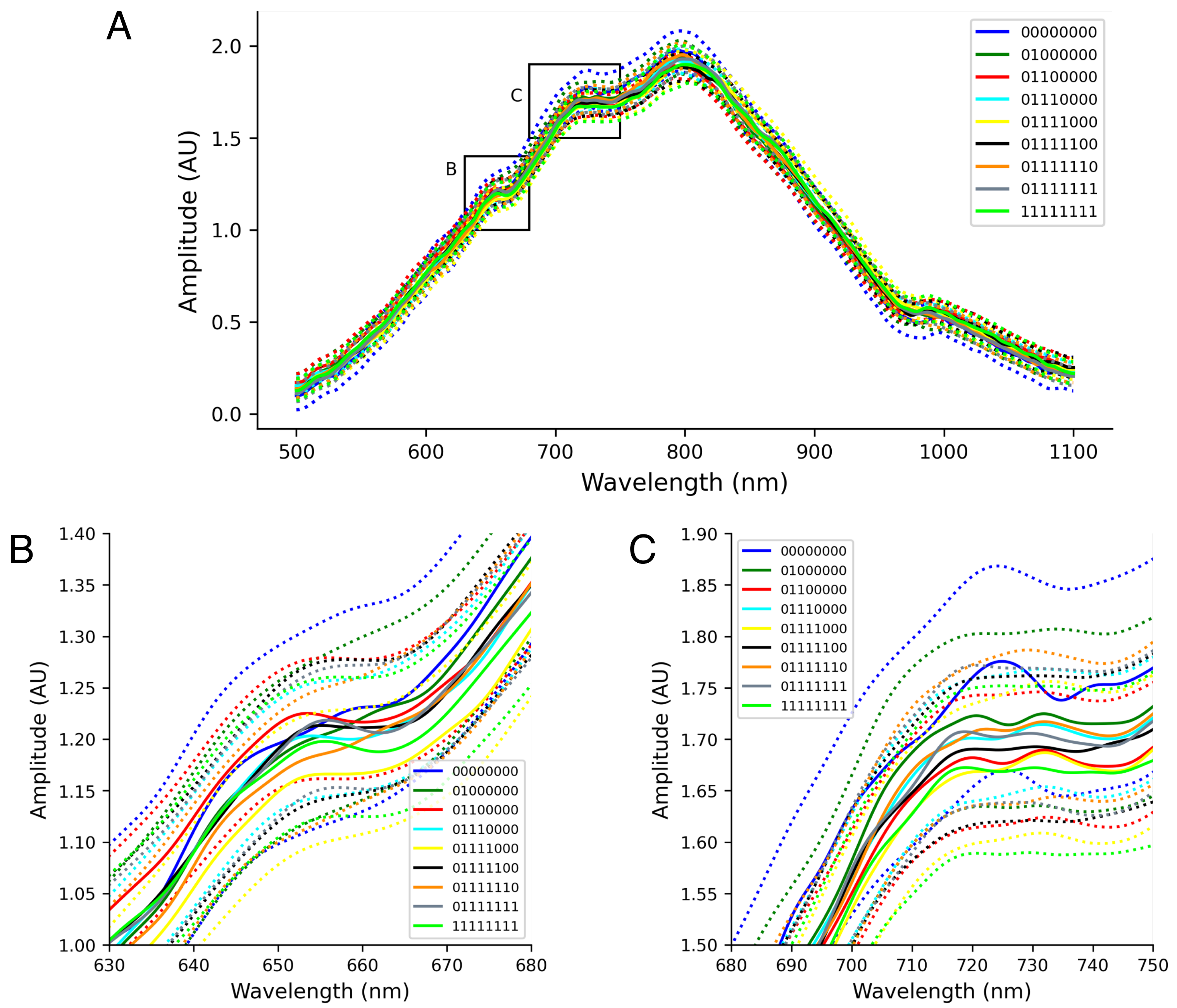

Figure S4. Spectra for training for assessment of volume fraction. (A) Mean (bold line) and standard deviation (dotted line) of spectra. Spectra were normalized. The spectra also were filtered to improve visualization using a 1D gaussian kernel with standard deviation of 20 measures (~3.3 nm). (B, C) Zoom into marked regions in (A).

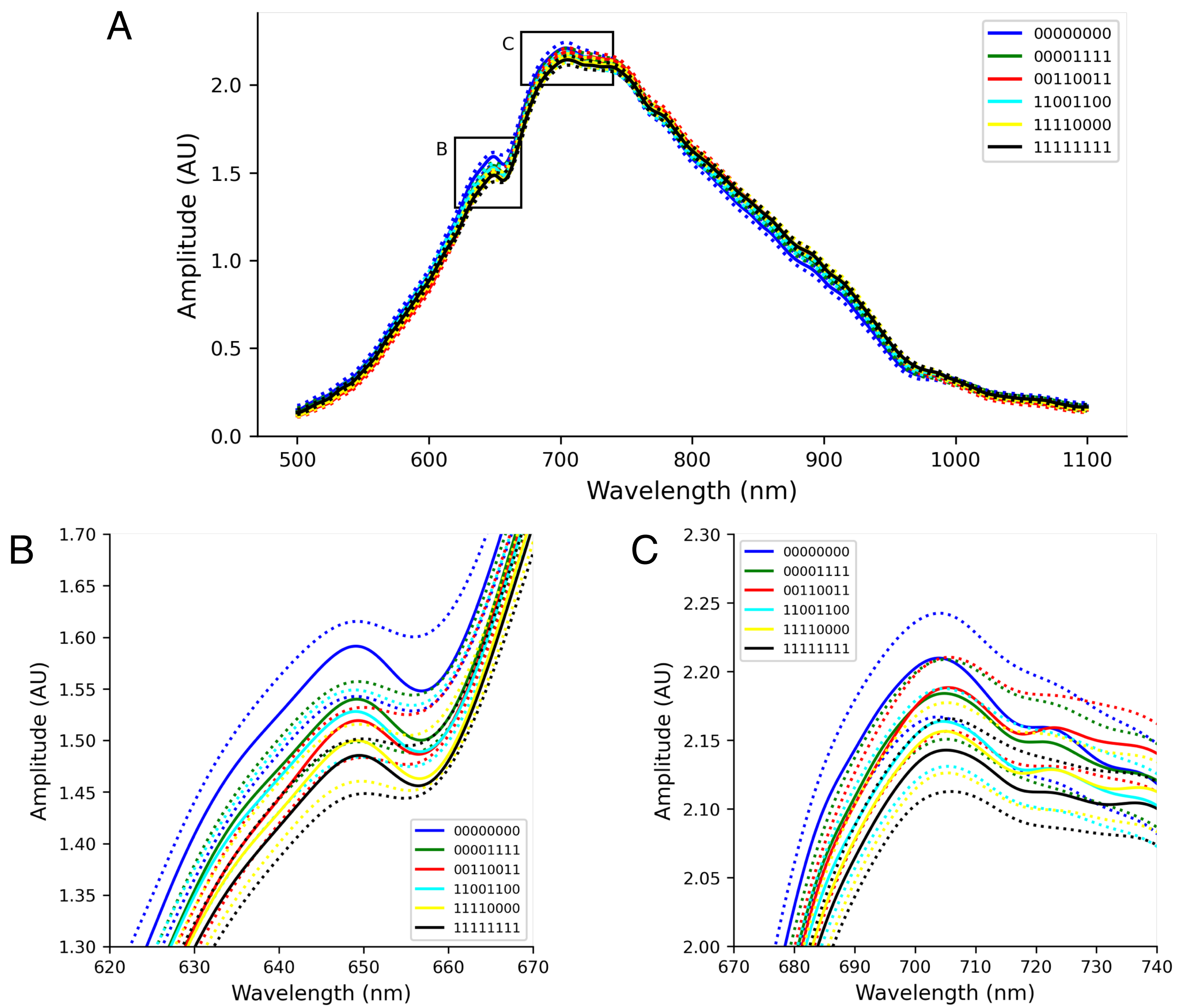

Figure S5. Spectra for training for assessment of permutated arrangements. (A) Mean (bold line) and standard deviation (dotted line) of spectra. Spectra were normalized. The spectra also were filtered to improve visualization using a 1D gaussian kernel with standard deviation of 20 measures (~3.3 nm). (B, C) Zoom into marked regions in (A).
